# Structural basis for distinct roles of SMAD2 and SMAD3 in FOXH1 pioneer-directed TGF-β signaling

**DOI:** 10.1101/gad.330837.119

**Published:** 2019-11-01

**Authors:** Eric Aragón, Qiong Wang, Yilong Zou, Sophie M. Morgani, Lidia Ruiz, Zuzanna Kaczmarska, Jie Su, Carles Torner, Lin Tian, Jing Hu, Weiping Shu, Saloni Agrawal, Tiago Gomes, José A. Márquez, Anna-Katerina Hadjantonakis, Maria J. Macias, Joan Massagué

**Affiliations:** 1 Institute for Research in Biomedicine (IRB Barcelona), The Barcelona Institute of Science and Technology, Barcelona 08028, Spain;; 2 Cancer Biology and Genetics Program, Memorial Sloan Kettering Cancer Center, New York, New York 10065, USA;; 3 Developmental Biology Program, Memorial Sloan Kettering Cancer Center, New York, New York 10065, USA;; 4 EMBL Grenoble, 38042 Grenoble Cedex 9, France;; 5 ICREA, 08010 Barcelona, Spain

**Keywords:** FOXH1, SMAD2, SMAD2 structure, SMAD3, TGF-β signaling, embryonic stem cell, mesendoderm differentiation, pioneer transcription factor

## Abstract

TGF-β receptors phosphorylate SMAD2 and SMAD3 transcription factors, which then form heterotrimeric complexes with SMAD4 and cooperate with context-specific transcription factors to activate target genes. Here we provide biochemical and structural evidence showing that binding of SMAD2 to DNA depends on the conformation of the E3 insert, a structural element unique to SMAD2 and previously thought to render SMAD2 unable to bind DNA. Based on this finding, we further delineate TGF-β signal transduction by defining distinct roles for SMAD2 and SMAD3 with the forkhead pioneer factor FOXH1 as a partner in the regulation of differentiation genes in mouse mesendoderm precursors. FOXH1 is prebound to target sites in these loci and recruits SMAD3 independently of TGF-β signals, whereas SMAD2 remains predominantly cytoplasmic in the basal state and set to bind SMAD4 and join SMAD3:FOXH1 at target promoters in response to Nodal TGF-β signals. The results support a model in which signal-independent binding of SMAD3 and FOXH1 prime mesendoderm differentiation gene promoters for activation, and signal-driven SMAD2:SMAD4 binds to promoters that are preloaded with SMAD3:FOXH1 to activate transcription.

## Abstract (precis)

In this study, Aragon et al. set out to address how signal-driven SMAD transcription factors activate differentiation genes. Their results support a model in which signal-independent binding of SMAD3 and FOXH1 prime mesendoderm differentiation gene promoters for activation, and signal-driven SMAD2:SMAD4 binds to promoters that are preloaded with SMAD3:FOXH1 to activate transcription.

Transforming growth factor β (TGF-β) signaling is crucial for metazoan development, tissue homeostasis, wound healing, and immune surveillance (David and Massagué 2018). Malfunctions of TGF-β signaling cause developmental defects, immune disorders, fibrosis, and cancer. TGF-β and other cytokines in this family signal through receptor serine/threonine kinases that phosphorylate SMAD transcription factors at C-terminal residues. TGF-β, Nodal, and Activin receptors phosphorylate SMAD2 and SMAD3. Thus activated, SMAD2 and SMAD3 form complexes with SMAD4, accumulate in the nucleus, and recruit coactivators and repressors to regulate the expression of target genes.

Although SMAD proteins have intrinsic DNA-binding activity, their binding to target regulatory regions requires other transcription factors as DNA-binding partners, as observed in progenitor cells of diverse lineages (Chen et al. 1997; Germain et al. 2000; Hata et al. 2000; Qing et al. 2000; Seoane et al. 2004; Mullen et al. 2011; Trompouki et al. 2011). As a result, cells interpret TGF-β signals in a context-dependent manner, which is partly dictated by cooperating lineage-restricted transcription factors (David and Massagué 2018). In embryonic stem cells these SMAD partners include the forkhead factor FOXH1 (previously known as FAST1), which is essential for Nodal TGF-β signals to activate mesendoderm differentiation genes during vertebrate gastrulation (Chen et al. 1997). FOXH1 and other forkhead family members are pioneer factors that can bind to condensed chromatin and prime-specific loci for recruitment of additional transcription factors (Iwafuchi-Doi and Zaret 2016; Charney et al. 2017). FOXH1 also binds directly to a conserved region of SMAD2 and SMAD3 (Liu et al. 1997). Whether SMAD proteins interact with their DNA-bound partners in the basal state, and how SMAD2 and SMAD3 individually function in this context, remain as open questions.

SMAD proteins consist of an N-terminal DNA-binding domain (MH1 domain) and a C-terminal region including the linker and the MH2 domain that contacts partner transcription factors like FOXH1, coactivators, and corepressors (Shi and Massagué 2003; Aragon et al. 2011; Macias et al. 2015; Miyazono et al. 2018). In vertebrates, SMAD2 and SMAD3 are coexpressed in most cell types and have similar amino acid sequences except for a unique highly conserved 30-amino acid insert, called the E3 insert, in the MH1 domain of SMAD2 and encoded by an alternatively spliced exon. The SMAD2β isoform lacking the E3 insert is a minor species in most tissues.

The SMAD2 E3 insert gained notoriety when it was shown that recombinant SMAD3, SMAD4 and other SMAD proteins bound to DNA in vitro, whereas SMAD2 containing this insert did not (Dennler et al. 1998; Zawel et al. 1998). These observations led to the long-standing albeit paradoxical notion that SMAD2, a crucial mediator of TGF-β transcriptional responses, does not bind DNA. Also associated with the E3 insert is the ability of SMAD2 to remain predominantly monomeric as it shuttles between the cytoplasm and the nucleus in the absence of TGF-β signals (Jayaraman and Massagué 2000; Xu et al. 2002). In contrast, SMAD3 moves more readily into the nucleus (Kurisaki et al. 2001) and is engaged in macromolecular complexes even without TGF-β inputs (Jayaraman and Massagué 2000; Liu et al. 2016). Mouse *Smad2* and *Smad3* knockouts have different phenotypes (Nomura and Li 1998; Zhu et al. 1998; Ashcroft et al. 1999; Datto et al. 1999; Heyer et al. 1999; Dunn et al. 2004, 2005). Despite these differences, SMAD2 and SMAD3 are frequently studied with cross-reactive reagents, referred to as “SMAD2/3” in the literature, and treated as functionally equivalent proteins.

Here we demonstrate that SMAD2 binds DNA, define the role of the E3 insert, and elucidate individual functions of SMAD2 and SMAD3 in the regulation of mesendoderm differentiation genes. We observe that properly folded SMAD2 protein has intrinsic DNA-binding activity, which is modulated by the ensemble of conformations adopted by the E3 insert in solution. Using isoform-specific SMAD knockouts in mouse embryonic stem cells (ESCs) and mesendoderm progenitors, we show that SMAD2 occupies regulatory regions in mesendoderm differentiation genes only in the presence of TGF-β Nodal signals. In contrast, SMAD3 is recruited to these regions by FOXH1 under basal conditions without TGF-β signaling, and this complex is joined by SMAD2 and SMAD4 in response to TGF-β signals. The distinct behavior of SMAD2 is imparted by the E3 insert and is important for mesendoderm differentiation. These insights suggest a model in which SMAD2 acts as a classic receptor-activated signal transducer, whereas SMAD3 and FOXH1 bound to differentiation gene loci under basal conditions prime these sites for the incorporation of signal-driven SMAD2 and SMAD4 and transcriptional activation.

## Results

### DNA-binding activity of SMAD2

**Figure 1.**
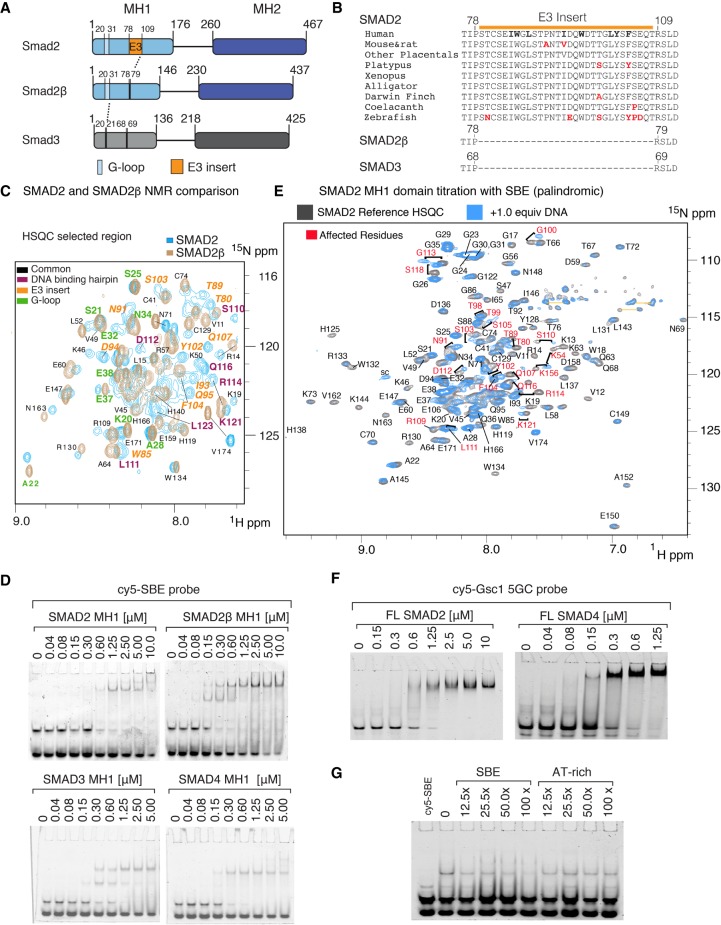
SMAD2 binding to DNA. (*A*) Schematic representation of SMAD2, 2β, and SMAD3 proteins. (*B*) Sequence conservation of the SMAD2 E3 insert. Aromatic and hydrophobic residues are bolded in the human sequence. Nonidentical residues are highlighted in red. Human SMAD2β and SMAD3 are included for comparison. (*C*) Overlay of ^1^H,^15^N-HSQC region (full experiment shown as SF1D) recorded at 600 MHz, SMAD2 in blue, SMAD2β in beige. Some residues are labeled and color-coded by region. (*D*) Native polyacrylamide gel electrophoresis mobility shift assays (EMSA) with the indicated concentrations of human SMAD MH1 domains and cy5-labeled SBE probe. (*E*) MH1 domain binding to DNA using nuclear magnetic resonance (NMR). Residues affected upon addition of the DNA are labeled in red. Unaffected residues are labeled in black. (*F*) EMSA with the indicated concentrations of full-length SMAD2 and SMAD4 proteins and cy5-labeled Gsc1 5GC probe. (*G*) EMSA with SMAD2 MH1 protein, cy5-SBE probe, and the indicated molar excess of unlabeled SBE probe or a nonbinding AT-rich probe.

SMAD2 and SMAD3 are similar in amino acid sequence (91% identity) (Supplemental Fig. S1A) except for a 10-residue extension of the loop connecting the first two α-helices, and the 30-amino acid E3 insert, which is spliced in SMAD2β, an isoform that closely resembles SMAD3 (Fig. 1A). The sequence of the E3 insert is highly conserved throughout vertebrate evolution (Fig. 1B) and located immediately N-terminal to the β2–β3 hairpin, the DNA-binding structure in SMAD MH1 domains (Shi et al. 1998). SMAD2 is vastly prevalent over SMAD2β at the mRNA level in most mouse tissues except the brain (ENCODE consortium) (Supplemental Fig. S1B).

Recombinant forms of SMAD2 and the isolated SMAD2 MH1 domain produced in mammalian cells or *E. coli* were reported to lack DNA-binding ability (Zawel et al. 1998; Dennler et al. 1999; Yagi et al. 1999). However, we found that the presence of N-terminal fusion tags as well as the protein expression and purification conditions markedly affected the solubility of recombinant SMAD2 MH1 domain expressed in *E. coli*. A previously used N-terminal fusion of glutathione S-transferase (GST) in SMAD2 (Zawel et al. 1998) yielded aggregated protein in our hands, even after cleavage of the GST portion. NMR analysis of these samples using ^1^H-^15^N heteronuclear single quantum correlation (HSQC) spectroscopy indicated poor signal dispersion of the amide resonances (Supplemental Fig. S1C) in contrast to the well-dispersed signals of SMAD4 MH1 domain used for comparison (Martin-Malpartida et al. 2017). Using 1D ^1^H-NMR to screen for optimal conditions, we found that a short cleavable N-terminal hexa-histidine tag, protein expression at 20°C, and mild lysis conditions in the presence of reducing agents and 10% glycerol yielded folded recombinant human SMAD2 and SMAD2β MH1 domains that were suitable for DNA binding and structural studies. The presence of tertiary structure was also evident in the dispersion of the amide resonances of both SMAD2 and SMAD2β splicing variants (Supplemental Fig. S1D). Using ^13^C-^15^N-^2^H labeled samples and triple resonance NMR experiments we were able to identify most of the backbone resonances for both SMAD2 and SMAD2β MH1 domains, including the residues corresponding to the E3 insert in SMAD2 (Fig. 1C). Under these conditions, the SMAD2 and SMAD2β folded proteins were monomers in solution, as determined by a combination of size exclusion chromatography and multiangle light scattering analysis (SEC-MALS) (Supplemental Fig. S1E). The same monomeric behavior was detected for SMAD3 MH1 domain used for comparison. The thermal stability analysis of these three samples showed that the SMAD2 melting temperature was 4°C and 6°C higher than those of SMAD3 and SMAD2β, respectively, with or without cognate DNA oligonucleotides (Supplemental Fig. S1F).

To compare the DNA-binding capacity of recombinant SMAD2, SMAD2β, SMAD3, and SMAD4 MH1 domains, we used nondenaturing electrophoretic mobility shift assays (EMSA) with fluorescent dsDNA oligonucleotides. These probes included the palindromic GTCT-AGAC SMAD-binding element (SBE) sequence (Zawel et al. 1998), its GTCTG variant, and the GGCTG and GGCGC sequences (5GC motifs) found in many SMAD target genes, including the mesendoderm differentiation gene *goosecoid* (*Gsc*) (Martin-Malpartida et al. 2017). The recombinant human and zebrafish SMAD2 MH1 domains demonstrated an affinity for a cy5-labeled SBE probe in the range of from 0.3 to 1.25-µM concentrations (Fig. 1D; Supplemental Fig. S1G), whereas the recombinant human SMAD2β, SMAD3, and SMAD4 MH1 domains bound this probe in the 0.1–0.6 µM concentration range (Fig. 1D). The addition of 1.0 molar equivalent of DNA probe to ^15^N labeled SMAD2 MH1 protein induced chemical-shift differences in residues located in and around the β2–β3 hairpin as well as in residues of the E3 insert, supporting the interaction observed in the EMSA assays (Fig. 1E).

The SMAD2 MH1 domain and full-length proteins also bound to different 5GC probes (Gsc1 and Gsc2 probes) from the *Gsc* promoter in the 0.6–1.2 µM concentration range (Fig. 1F; Supplemental Fig. S1H), whereas the full-length SMAD4 protein bound the Gsc1 probe at values between 0.15 and 0.30 µM (Fig. 1F). Further, the binding of SMAD2 to the SBE probe was inhibited by inclusion of unlabeled SBE oligonucleotide in the binding reaction at high molar excess, but not by inclusion of a nonspecific oligonucleotide (Fig. 1G). Thus, well-folded SMAD2 MH1 domain binds to SBE and 5GC probes specifically, albeit with threefold lower affinity than those of SMAD2β, SMAD3, and SMAD4 MH1 domains.

### X-ray crystal structure of SMAD2β MH1 domain bound to DNA

**Figure 2.**
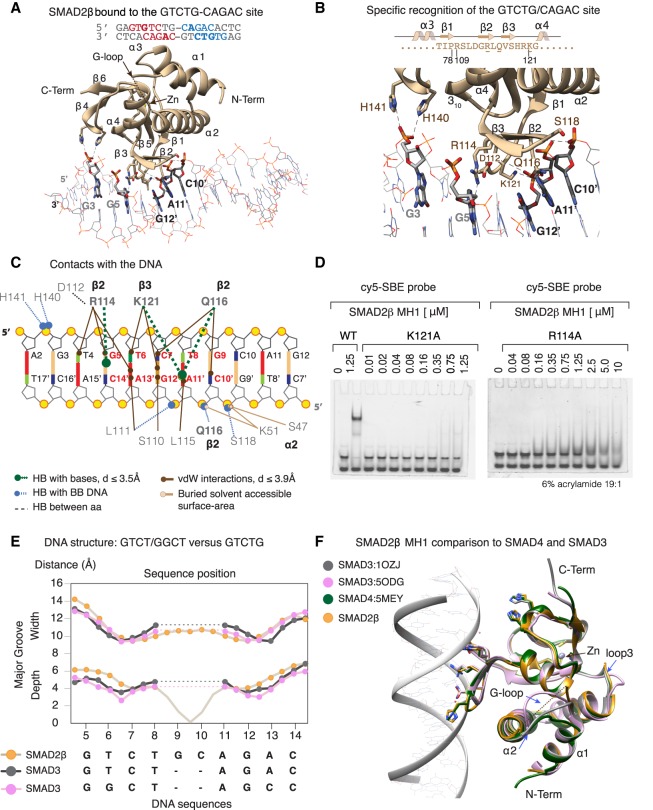
X-ray crystal structure of the SMAD2β MH1 domain bound to DNA. (*A*) Model structure of SMAD2β MH1 domain (beige) bound to the SBE motif (gray), refined at 2.7 Å resolution. Elements of secondary structure, residues that interact with DNA or that coordinate a Zn atom are indicated. The entire ASU is shown as Supplemental Figure S1A. (*B*) Close view of the binding site, with residues and bases involved in hydrogen bonds labeled. The stereo view representation of the electron density contoured at 1σ level (2Fo-Fc) is shown as Supplemental Figure S2B. The DNA-binding hairpin sequence and the residue numbering based on the SMAD2 sequence are indicated. (*C*) Schematic representation of the intermolecular protein–DNA contacts. Dashed lines indicate hydrogen bonds (HB) color-coded by interaction type. Solid lines indicate residues involved in van der Waals interactions or in reducing the solvent accessible area of the DNA as determined with DnaproDB (Sagendorf et al. 2017). (*D*) EMSA with two SMAD2β point mutations (R116 and at K121) and the cy5-labeled SBE probe kept at 7.5-nM concentration. The mutants showed a negligible ability to interact with DNA. 1D NMR experiments showing that the samples are properly folded are shown in Supplemental Figure S2F. (*E*) DNA shape comparison of SMAD2β bound to GTCTG site (this work, PDB: 6H3R), SMAD3 bound to GTCT or GGCT sites (PDB entries:1OZJ and 5ODG). Major groove width (*top*) and depth (*bottom*) were calculated using Curves+ (Lavery et al. 2009). Since the GTCT and GGCT sites are shorter than the GTCTG site, the gaps in the palindromic sequence are indicated as dashed lines. (*F*) Comparison of SMAD3 (graphite and orchid ribbons) and SMAD4 (*green*) MH1 complexes to that of SMAD2β (gold) bound to GTCTG site. All MH1 domains are very similar. The differences are observed in two loops (loop1 or G-loop and loop3) as well as at the length of helix α2 (indicated by an arrow). DNA shown is that of the SMAD2β structure (white ribbon).

To characterize the DNA-binding interaction of SMAD2 isoforms, we screened several oligonucleotide duplexes containing either 5GCs or the 5-bp SBE GTCTG motif. The best diffracting crystals were obtained with an 18-bp dsDNA containing the palindromic GTCTG sequence and SMAD2β (2.75 Å resolution), whereas the crystals obtained with SMAD2 MH1 protein could not be optimized to diffract below 5 Å resolution. The SMAD2β MH1-DNA complex was solved by molecular replacement using a model derived from SMAD3 (PDB ID: 5ODG) and refined to final Rwork/Rfree values of 20.1% and 22.2%, respectively. The overall structure of the complex is well defined in the electron density map, with the asymmetric unit (ASU, space group P4_3_2_1_2) containing two SMAD2β MH1 monomers and one dsDNA (Fig. 2A; Supplemental Fig. S2A,B). The final model contains the 18-bp DNA, and the amino acids 8–170 in chains A and B, with more than 97% of the residues lying in the most favored regions of the Ramachandran plot (statistics shown in Supplemental Table S1). To facilitate the structural comparison of both SMAD2 isoforms we numbered the SMAD2β MH1 domain according to SMAD2 sequence (Fig. 2B).

Like the reported MH1 domain structure of other SMAD proteins (SMAD1, SMAD3, SMAD4, and SMAD5) (Shi et al. 1998; BabuRajendran et al. 2010, 2011; Martin-Malpartida et al. 2017), the SMAD2β MH1protein fold is defined by four α-helices forming a bundle, a 3_10_ helix, and three anti-parallel pairs of short strands (β1–β5, β2–β3, and β4–β6). The structure is stabilized by a Zn^2+^, as indicated by a strong electron density in the proximity of cysteines 74,149, and 161 and histidine 166 (Fig. 2A; Supplemental Fig. S2C). The loop connecting the α1 and α2 helices (G-loop, Supplemental Fig. S1A), was excluded in the refined model because it is not well defined in the electron density map. This loop is longer than in other SMADs and contains ten extra residues (SAGGSGGAGG) compared with SMAD3. The flexibility of this loop was confirmed by low ^1^H,^15^N heteronuclear NOE values (Supplemental Fig. S3A) and by the presence of partially overlapped amides as indicated in the ^1^H-^15^N HSQC (Fig. 1D; Supplemental Fig. S1D).

The DNA-binding region includes the convex face of the β2–β3 hairpin (Fig. 2B, residues 79–109, highlighted in beige) and the short loop connecting the β1 and β2 strands. The β2–β3 hairpin contains three conserved residues, Arg114, Gln116, and Lys121, which participate in a network of hydrogen bonds that define specific interactions with the DNA major groove. We also detected hydrogen-bond interactions between the Ser118, Leu111, Gln116 (backbone), and His140 and His141 (side-chains) with C10′, G12′, A11′, and G3 bases. In the complex, the MH1 domain covered seven base pairs, from G3 to G9 (Fig. 2B,C). The complex interface is further stabilized by the electrostatic charge complementarity at the binding interface and by a set of van der Waals interactions between Leu111, Ser110, and Leu115 and the DNA, as measured by DNAproDB (Fig. 2C; Sagendorf et al. 2017). Binding to DNA was also corroborated by NMR HSQC titrations (Supplemental Fig. S2E). The role of Arg114 and Lys121 in binding to DNA was verified with SMAD2β MH1 proteins containing Arg114Ala and Lys121Ala point mutations. These mutations strongly diminished the affinity for DNA (Fig. 2D) without affecting the overall structure of SMAD2β (Supplemental Fig. S2F).

The main features of the SMAD2β MH1:GTCTG complex are similar to those of SMAD3 (PDB entries: 1OZJ and 5ODG) and SMAD4 (PDB entries: 3QSV and 5MEY) bound to GTCT and GGCT motifs. The main differences are concentrated at the DNA structure. The topological analysis of bound DNA using Curves (Fig. 2E; Lavery et al. 2009) revealed that the major groove is slightly wider and deeper in this SMAD2β/GTCTG-CAGAC complex than in the other complexes determined so far using the GTCT-AGAC motif. At the protein level, the similarity includes the conserved pattern of base-amino-acid interactions and the overall structure of their MH1 domains. This similarity is manifested in the RMSD values of the Cα superimpositions (0.4 Å for SMAD3/SMAD2β and 0.5 Å for SMAD4/SMAD2β). As observed in the superimposition of structures, most differences in SMAD MH1 domains concentrate in loops, particularly within the first loop and in the length of the α2 helix, which is one turn longer in SMAD2 and SMAD3 than in SMAD4 (Fig. 2F).

### Conformational analysis of the SMAD2 E3 insert and its role in DNA binding

Next, we investigated the structural properties of the SMAD2 MH1 domain (174 aa) using heteronuclear multidimensional NMR spectroscopy and small-angle X-ray scattering (SAXS). For comparison, we also acquired the same experiments for the SMAD2β construct (144 aa) used in the X-ray crystallographic structures. To analyze the flexibility of these structures in solution, we measured longitudinal and transverse relaxation times (*T1* and *T2*) as well as heteronuclear 2D ^1^H-^15^N-nuclear Overhauser effect (hetNOE) in the absence of DNA.

**Figure 3.**
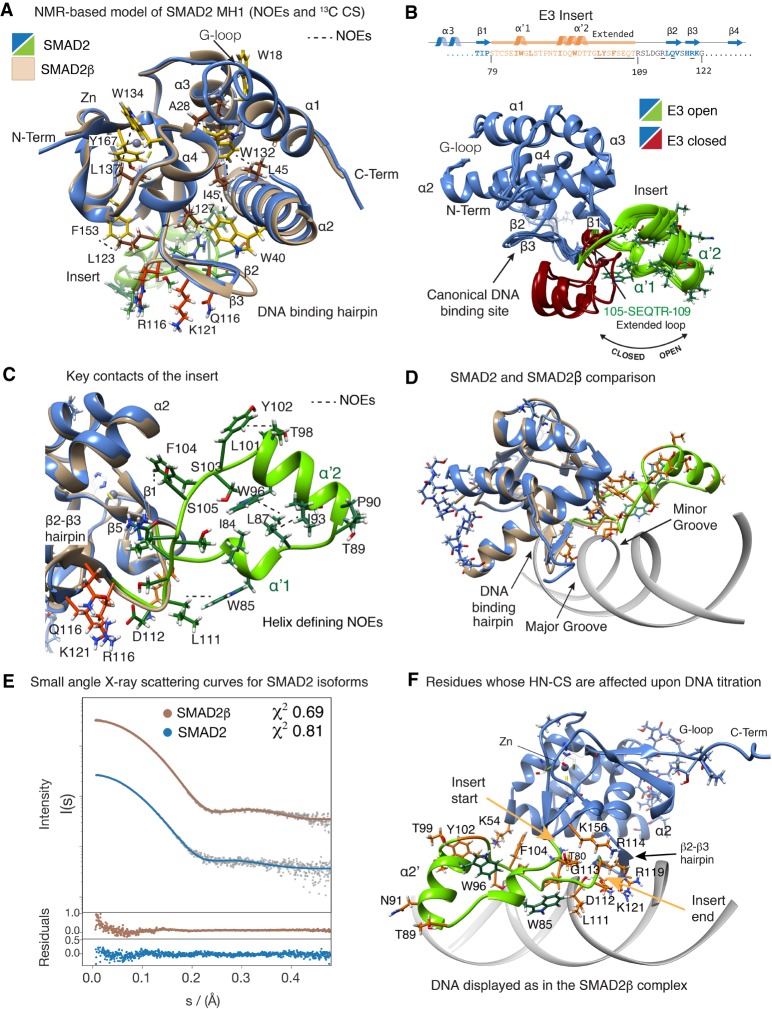
SMAD2 MH1 conformations in solution. (*A*) Overlay of the SMAD2β MH1 domain (beige) to different NMR-based models of the SMAD2 MH1 domain (blue) determined using NMR restrains and pyRosetta. Contacts used to determine the MH1 fold are labeled and shown in yellow (aromatic residues), brown (hydrophobic residues), and red (DNA-binding hairpin). The elements of secondary structure were determined based on ^13^C chemical shifts and NOEs. The MH1 core is shown in blue and the E3 insert is depicted in chartreuse. Observed NOEs are represented as dashed lines. (*B*) The sequence of the E3 *insert* (orange) and the elements of secondary structure are schematically indicated at the *top*. Residues affected upon DNA binding are underlined. Different orientations of the E3 are shown (open in chartreuse, closed in dark red. Conformations were calculated as described in the text. (*C*) Key features of the E3 *insert*. Secondary structure elements (chartreuse) were determined by the α, HN_(1, 1+3)_ pattern of NOEs and by ^13^C values. Residues involved in packing of the helices are shown in dark green and are labeled. Contacts are indicated by a dashed line. (*D*) Overlay of the NMR SMAD2 open conformation (blue) and SMAD2β (PDB:6H3R, beige) complex. In the SMAD2 open conformation the β2-β3 DNA-binding hairpin is accessible. (*E*) Small angle X-ray scattering (SAXS) analysis of the SMAD2 (*blue*) and SMAD2β (*beige*) MH1 domains. Experimental and graphical output of the best fit are shown for each protein. Residuals for the fittings are shown *below* the data. (*F*) SMAD2 MH1 open conformation (blue) superimposed to the DNA as bound in the SMAD2β complex. Residues displaying chemical-shift changes are indicated in orange and labeled. Contacts with the major groove are conserved in both isoforms. The “SEQTR” fragment present in the E3 *insert* only, lies in the proximity of the minor groove. The starting and ending points of the E3 insert are indicated. A 90° rotation is shown as Supplemental Figure S3B.

The analysis of the 3D NMR datasets allowed us to complete the assignment of the backbone resonances of most residues in both SMAD2 isoforms. The differences of the Cα and Cβ chemical shifts relative to reference values, together with the analysis of the 3D ^15^N edited-NOESY data, corroborated that in solution both isoforms show the presence of the characteristic structural elements of a MH1 fold, including four helices, six strands, and bound Zn^2+^. We were able to unambiguously assign several NOEs between key aromatic and hydrophobic residues that corroborate the packing of each MH1 domain (Fig. 3A). Furthermore, the common residues for both isoforms display highly similar Cα and Cβ chemical shifts, suggesting that the presence of the E3 insert does not perturb the main features of the MH1 structure (Supplemental Fig. S3A). Analysis of the ^1^H-^15^N heteronuclear Overhauser effect (NOE) experiments corresponding to the SMAD2 isoform indicated that the DNA-binding hairpin and the insert are flexible. The SMAD2β isoform (lacking the E3 insert) also has a flexible DNA-binding hairpin, as previously observed in the SMAD4 MH1 domain (Supplemental Fig. S3A; Martin-Malpartida et al. 2017). This flexibility facilitates the interaction with slightly different DNA sequences including the SBE and 5GC motifs.

In the E3 insert, the Cα and Cβ chemical shifts, the pattern of NOEs, and their intensities as well as the heteronuclear NOE values revealed that the insert populates an equilibrium of conformers. These conformers contain two short helical segments (residues 83–86 and 91–98, respectively) connected to the MH1 domain structure by two loops, the first loop located adjacent to the β1 strand and the second preceding the β2–β3 hairpin (DNA-binding hairpin). Considering the flexibility of the E3 insert, the boundaries of these helical turns vary slightly between different conformers. We identified NOE contacts between residues located in the E3 insert but not between these residues and the rest of the protein, suggesting that the E3 insert is attached to the MH1 domain without perturbing its compact fold. This is consistent with the close similarity observed in the overlay of HSQC data for SMAD2 and SMAD2β, and the comparison with the SMAD2β structure (Supplemental Fig. S1D; Supplemental Movie S1).

To obtain a 3D description of these conformers in solution we generated a set of 100 templates using PyRosetta software (Chaudhury et al. 2010). For these templates, we leveraged the crystal structure of SMAD2β, the structural restrictions obtained from the NMR backbone assignments, as well as the unambiguously assigned distance restraints derived from the SMAD2 NOESY data including residues located at the E3 insert (Fig. 3A–C). Each model was later refined with the FastRelax protocol. In this refinement, the domain had to fulfill all experimental restraints to maintain the MH1 domain fold, whereas the E3 insert was allowed to move and readjust the helical boundaries. This approach generated a family of NMR-based models with the E3 insert adopting open and closed conformations with respect to the MH1 domain that satisfy the secondary structure and intrainsert NOEs restraints (shown in green and dark red, respectively, Fig. 3B). Furthermore, the E3 insert conformations were further corroborated by analyzing the small-angle X-ray scattering (SAXS) profiles . The SAXS data obtained for SMAD2 and SMAD2β MH1 domains indicated a radius of gyration (Rg) of 19 and 17 Å and a maximum distance (Dmax) of 74 and 66 Å, in agreement with two MH1 domains that differ in size (174 and 144 residues, respectively) (Fig. 3E). The analysis also indicated that open conformations are more abundant than closed ones, (70:30 ratio) according to the models that satisfy the experimental curves. These conformations observed by SAXS support the large conformational flexibility of the E3 insert revealed by the backbone relaxation experiments. The SAXS data also supports the conformational variability sampled by the G-loop, in agreement with the faster motions detected by NMR, with heteronuclear NOE values below 0.3 (Fig. 3B; Supplemental Fig. S3B).

For a given conformation, the packing of the two short helices is stabilized by a network of interactions between aromatic and hydrophobic residues (Fig. 3C; Supplemental Fig. S3C). One of the key residues for these interactions, Trp96, shows abundant NOEs with Ile84, Leu87, Thr92, and Leu101. Phe102 also shows NOEs with Leu101 and with Lys51. In contrast, Trp85 is highly exposed to the surface, and shows NOEs with only neighbor residues (Ile84 and Ser82).

In the NMR titrations performed with an oligo containing the GTCTG/CAGAC site we observed chemical-shift differences at residues located at the β2–β3 hairpin as well as at the 105-SEQTR-109 residues preceding the hairpin and in T99, G100, Y102, and F104, located at the last helical turn of the E3 insert. All these residues in the hairpin and in the 105–109 region are located in the proximity of both major and minor DNA grooves, as shown in the superposition of the SMAD2 model to the SMAD2β complex (Fig. 3D,F). In addition, the presence of DNA also induced chemical-shift perturbations at the C-term part of α2 helix and at residues located at the loop7. These perturbations might reflect the presence of part of the insert in the proximity of loop7 in the open conformation stabilized upon DNA binding. As observed in SMAD2β, the interaction with DNA in EMSA experiments was inhibited by a double mutation Arg114Ala and Lys121Ala, (even though this mutant protein was well folded, Supplemental Fig. S3D,E) but not by single mutations as with SMAD2β isoform, corroborating the direct implication of the SMAD2 β2–β3 hairpin and of the insert in DNA binding.

Collectively, these results indicate that SMAD2 is a conformationally dependent DNA-binding protein, with this binding activity conditioned by the different conformations adopted by the E3 insert. Thus, the ensemble of conformations occluding the DNA-binding site would reduce the effective number of molecules able to interact with DNA (Supplemental Figure S3F). This feature may explain the higher concentrations of SMAD2 protein required for a similar shift of DNA probes in EMSA assays compared with SMAD2β, SMAD3, or SMAD4 (refer to Fig. 1D–G). Moreover, the dynamic properties of the E3 insert and the presence of solvent exposed hydrophobic and aromatic residues in the insert suggest a basis for the propensity of recombinant SMAD2 proteins to precipitate and lose DNA-binding activity during purification and storage.

### SMAD2, SMAD2β, and SMAD3 in mESCs

The finding that SMAD2 binds DNA and the E3 insert conditionally auto-inhibits this activity raised the question of whether the E3 insert restrains or enhances signaling in TGF-β pathway. To investigate this question, we focused on mouse embryonic stem cells (mESCs; line ES-E14TG2a.4, ATCC), which undergo Nodal-dependent mesendoderm differentiation when placed in differentiation-permissive suspension cultures (absence of leukemia inhibitory factor, LIF) (Nishikawa et al. 1998; Xi et al. 2011). Under these conditions, mESCs form embryoid bodies (EBs) containing mesendoderm progenitors that progressively express differentiation genes over a 4-d period. Differentiation is driven by autocrine Nodal and can be accelerated by addition of exogenous Activin A (an available ligand for Nodal/Activin receptors), recapitulating signaling and transcriptional events that occur in the embryo at gastrulation (Wang et al. 2017).

**Figure 4.**
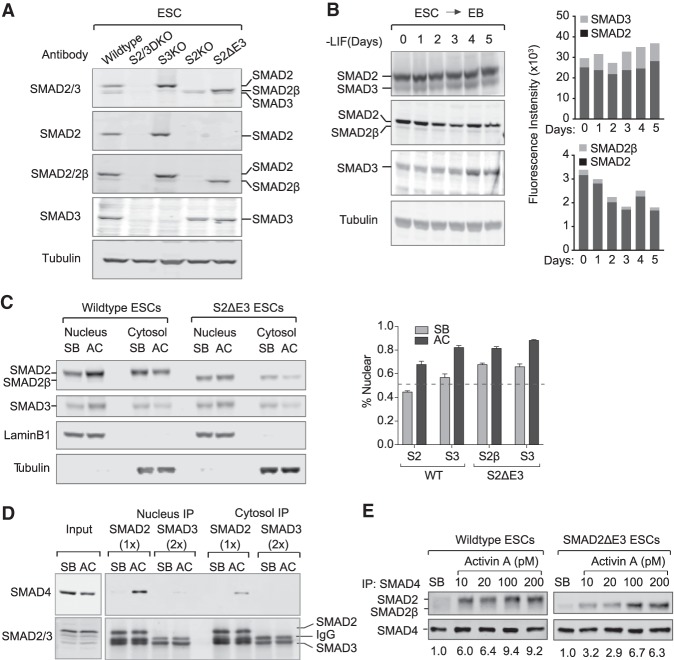
SMAD2, SMAD2β, and SMAD3 in mESCs. (*A*) Western immunoblotting analysis of SMAD2, SMAD2β, and SMAD3 in the indicated mESC lines, using antibodies of the indicated specificity. Tubulin was used as a loading control. (*B*) Immunoblotting analysis of SMAD2, SMAD2β, and SMAD3 in wild-type mESCs and derived EBs. Cells were collected at indicated time point after LIF removal to allow EB formation. (*Right*) Plot of fluorescence intensity of the SMAD2, SMAD3, and SMAD2β bands determined using an Odyssey imaging system. (*C*) Immunoblotting of SMAD2, SMAD2β, and SMAD3 of cytosolic and nuclear fractions from wild-type and S2ΔE3 mESCs incubated with SB431542 (SB) for 6 h or Activin A (AC) for 1.5 h. Lamin B1 and tubulin were used as loading control for nuclear and cytosolic fractions. (*Right*) Plot of fluorescence intensity of the nuclear and cytosolic bands determined using an Odyssey imaging system and percentage of nuclear immunofluorescence for each sample. (*D*) mESCs were incubated with SB for 6 h or Activin for 1.5 h and fractionated into nuclear and cytosolic fractions. Anti-SMAD4 and anti-SMAD2/3 immunoblotting of aliquots from these samples (*input*) or of anti-SMAD2 and anti-SMAD3 immunoprecipitates was performed to determine the levels of SMAD2-bound and SMAD3-bound SMAD4. (*E*) Signal-dependent interaction of SMAD2 and SMAD2β with SMAD4. Wild-type and S2ΔE3 mESCs were incubated with SB for 6 h, followed by a 2-h incubation with SB or the indicated concentrations of Activin. Anti-SMAD4 immunoprecipitates from these cells were subjected to anti-SMAD2/2β or anti-SMAD4 immunoblotting. The densities of SMAD2 or SMAD2β pulled down by SMAD4 were measured by Odyssey imaging system and marked *below* the immunoblotting.

We used CRISPR/Cas9 to generate mESCs that were *Smad2*^−/−^ (SMAD2 knockout, S2KO), *Smad3*^−/−^ (SMAD3 knockout, S3KO), *Smad2*^−/−^; *Smad3*^−/−^ (SMAD2 and SMAD3 double knockout, S2/3DKO), or deleted for *Smad2* exon 3 (S2ΔE3) (Fig. 4A; Supplemental Fig. S4A). The relative abundance of SMAD2, SMAD2β, and SMAD3 in the wild-type and mutant mESCs was determined by immunoblotting of cell lysates using a panel of isoform-specific antibodies as well as a cross-reactive anti-SMAD2/3 antibody (Fig. 4A,B). In wild-type mESCs, SMAD2 was more abundant than SMAD3, with a SMAD2:SMAD3 ratio of ∼6:1, as determined by anti-SMAD2/3 immunoblotting. The SMAD3 level increased during EB development, reaching a 4:1 SMAD2:SMAD3 ratio by day 3. SMAD2β was present in low abundance, with a SMAD2:SMAD2β ratio of ∼15:1. The *Smad2*:*Smad2β* transcript ratio was ∼20:1 in mESCs, as determined by paired-end RNA-seq read distribution of exon 3 transcripts (Supplemental Fig. S4B), which is similar to the transcript ratio reported in early mouse embryo (Peng et al. 2016).

We treated mESCs with the Nodal/Activin receptor inhibitor SB431542 (SB) to suppress endogenous Nodal activity (Supplemental Fig. S4C) or with Activin A (AC) to acutely activate the pathway (Supplemental Fig. S4D). Immunoblotting analysis of nuclear and cytoplasmic fractions in the absence of signaling showed that SMAD3 had a slightly more nuclear distribution than SMAD2 (56% nuclear SMAD3 versus 44% nuclear SMAD2), and Activin addition augmented the nuclear accumulation of both proteins (Fig. 4C). Immunoprecipitation of endogenous SMAD2 and SMAD3 and immunoblotting of these samples with anti-SMAD4 antibodies showed that SMAD2 accounts for most of the bound SMAD4 in Activin-treated mESC cells. No SMAD2–SMAD4 or SMAD3–SMAD4 interactions were detected in SB-treated cells (Fig. 4D). These results are in line with observations reported in human cells expressing exogenous SMAD proteins (Liu et al. 2016).

One possible reason for the low level of SMAD3:SMAD4 complex in Activin-treated ESCs is the fourfold lower abundance of SMAD3 relative to SMAD2 in these cells. To address this question, we performed experiments with S2ΔE3 mESCs, which express high levels of SMAD2β instead of SMAD2 from the endogenous *Smad2* locus (Fig. 4A). SMAD2β, which closely resembles SMAD3, showed a similar subcellular distribution as SMAD3, with a more nuclear distribution than SMAD2 under basal conditions (Fig. 4C). However, SMAD2β clearly bound SMAD4 (Fig. 4E). These results suggested that the E3 insert favors the cytoplasmic localization of SMAD2 and the formation of signal-induced SMAD2:SMAD4 complexes, whereas SMAD3 is more nuclear, and combined with the lower abundance of SMAD3, this limits the interaction of SMAD3 with SMAD4.

### FOXH1-dependent Nodal/Activin gene responses

**Figure 5.**
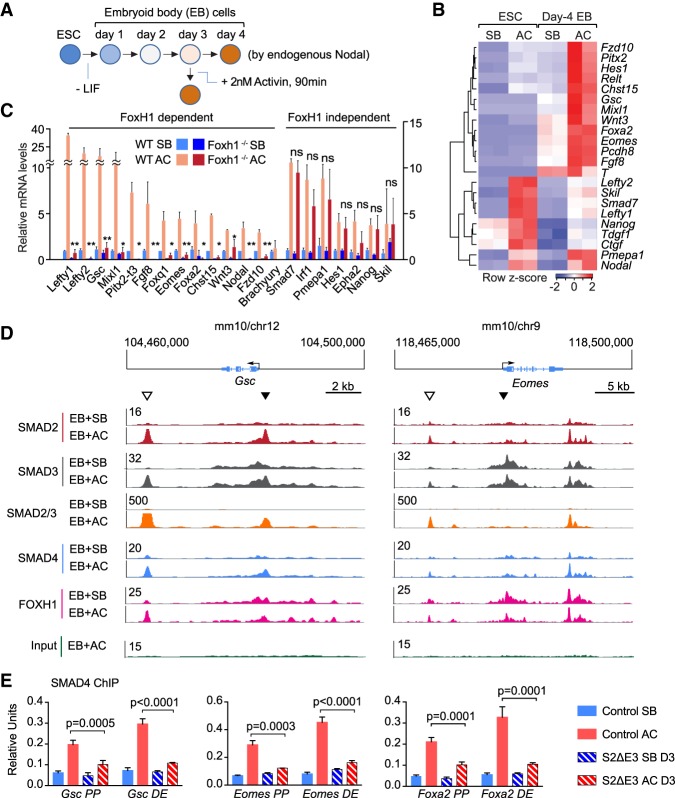
SMAD binding to FOXH1 pioneer-dependent mesendoderm genes. (*A*) Scheme of ES-E14TG2a.4 mESC differentiation into EBs rich in mesendoderm progenitors. Differentiation is enabled by placing of mESCs in media devoid of LIF. Starting on days 2–3, EB start expressing mesendoderm genes and losing expression of pluripotency genes. The process is driven by autocrine Nodal in a feedforward loop. Mesendoderm gene expression peaks on day 4, but day-3 EBs can be stimulated to acutely increase the expression of these genes by treatment with Activin A, which signals through Activin/Nodal receptors. Activin addition to cells in the ESC stage increases the expression of certain pluripotency genes (e.g., *Nanog*) and negative feedback genes (e.g., *Smad7*, *Skil*), but the cells are not yet competent to respond to Activin with mesendoderm gene expression (Wang et al. 2017) and refer to *B*. (*B*) Heatmap showing the expression of Activin-responsive genes in mESCs and day-3 EBs treated with SB431542 (SB) or Activin (AC) for 1.5 h and analyzed by RNA-seq (GSE70486). Two biological replicates per condition were analyzed. (*C*) FOXH1 dependence of Activin gene response. mRNA levels of select Activin-responsive genes were determined by qRT-PCR analysis of wild-type and *Foxh1*^–/–^ EBs treated with SB or AC. mRNA levels of each gene are expressed relative to the levels in WT cells under SB treatment. *N* = 3. Error bars, S.D. *P*-values were calculated by unpaired *t*-test, (*) *P* < 0.05; (**) *P* < 0.01; ns, not significant. (*D*) SMAD and FOXH1 ChIP-seq tags on the *Gsc* and *Eomes* loci. Gene track view for SMAD2, SMAD3, SMAD4, and FOXH1 ChIP-seq data in ESCs, and day-3 EBs treated with SB or AC. Precleared chromatin prior to primary antibody addition (*Input*) is also shown. Tag densities normalized to reads per million reads. Gene bodies are schematically represented at the *top* of each track set. Closed arrowheads, proximal promoter (PP) sites; open arrowheads, distal enhancer (DE) sites used in *E*. (*E*) ChIP-qPCR analysis of SMAD4 binding to the PP and DE regions of *Gsc*, *Eomes*, and *Foxa2* in wild-type (WT) and S2ΔE3 mESCs. *N* = 3; error bars represent SD, and *P*-values were calculated by *t*-test.

E14TG2a.4 mESCs start expressing mesendoderm differentiation genes 2–3 d after culture under differentiation conditions and reach peak expression of these genes on day 4. The process is dependent on autocrine Nodal, which is expressed at the ESC stage, and autocrine WNT3, which is progressively expressed over this time course (Wang et al. 2017). Activin addition to day-3 EBs induces the expression of these genes within 90 min, providing an assay for responsiveness to Nodal/Activin signals (Fig. 5A). Activin addition to day-3 EBs induced the expression of 22 genes, including mesendoderm differentiation genes (*Gsc*, *Eomes*, *Foxa2, T/Brachyury*, *Mixl1*, and others), and negative feedback mediators such as *Smad7* and *Skil,* as determined by RNA-seq analysis (Fig. 5B). Activin addition to mESCs under culture conditions that preserve pluripotency-induced negative feedback genes and pluripotency genes, but not mesendoderm differentiation genes (Fig. 5B). FOXH1 is essential for SMAD binding to and activation of mesendoderm differentiation genes (Chen et al. 1997). We used day-3 wild-type EBs and *Foxh1*^−/−^ EBs (Hoodless et al. 2001; Izzi et al. 2007) to determine the FOXH1 dependence of all Activin gene responses in this context. Real-time polymerase chain reaction (qRT-PCR) analysis of specific transcripts showed that a majority of mesendoderm differentiation genes required FOXH1 for induction by Activin, whereas other Activin target genes did not (Fig. 5C).

### Distinct patterns of SMAD interaction with FOXH1 target genes

FOXH1 functions as a pioneer factor that occupies regulatory regions in mesendoderm genes independently of Nodal inputs (Charney et al. 2017), whereas SMAD interactions with target loci are thought to depend on TGF-β signaling. We performed chromatin immunoprecipitation and sequencing (ChIP-seq) to analyze the interaction of SMAD2, SMAD3, SMAD4, and FOXH1 with *Gsc* and *Eomes* as representative FOXH1-dependent mesendoderm differentiation genes (Fig. 5D). FOXH1-binding motifs are present in the promoter regions of these genes (Charney et al. 2017; Martin-Malpartida et al. 2017). In day-3 EBs, FOXH1 ChIP tag peaks were present in these regions and more distal regions in the absence of Nodal signaling (SB treatment). The FOXH1 ChIP tag profile changed little upon cell treatment with Activin, consistent with FOXH1 acting as a prebound pioneer factor. The most conspicuous Activin-dependent change in the FOXH1 ChIP profile at these loci was an increased interaction with a *Gsc* downstream enhancer (Fig. 5D).

ChIP-seq analysis using a cross-reactive anti-SMAD2/3 antibody (refer to Fig. 4A) showed no signal in the *Gsc* and *Eomes* loci in the presence of SB, and strong signals at promoter and distal enhancer regions in response to Activin, as we previously reported (Wang et al. 2017). These distal enhancers are co-occupied by Tcf3 in response to Wnt3 in day-3 EBs. The SMAD4 ChIP pattern at these loci was similar to that observed with SMAD2/3 ChIP (Fig. 5D). The Activin-dependent increase in SMAD4 interaction with proximal and distal regions of *Gsc*, *Eomes*, and *Foxa2* was markedly blunted in S2ΔE3 cells compared with wild-type cells (Fig. 5E), indicating a superior ability of SMAD2 over SMAD2β to recruit SMAD4 to regulatory sites in mesendoderm genes in response to Nodal signals.

We also performed ChIP-seq analysis using isoform-specific anti-SMAD2 and anti-SMAD3 antibodies. The SMAD2 ChIP profile on the *Gsc* and *Eomes* loci resembled the SMAD2/3 ChIP profile and its dependence of Activin. In surprising contrast, the SMAD3 ChIP profile closely matched that of FOXH1 ChIP, including signal-independent interaction with the *Gsc* and *Eomes* promoters, Activin-induced interactions with the *Gsc* enhancer and, to a lesser extent, the *Eomes* upstream enhancer (Fig. 5D). The isoform-specific antibodies target MH1 domains of SMAD2 and SMAD3, whereas the anti-SMAD2/3 antibody targets the interdomain liker regions of SMAD2 and SMAD3. These ChIP results suggest that SMAD3 cobinds with FOXH1 to the *Gsc* and *Eomes* promoters in the absence of signal, and SMAD3 MH1 domain but not the linker region in this DNA-bound complex is exposed for antibody recognition.

### Preferential recruitment of SMAD3 by pioneer FOXH1

We tested the hypothesis that FOXH1 bound to regulatory regions of mesendoderm differentiation genes recruits SMAD3 preferentially over the more abundant SMAD2 in the absence of Nodal/Activin signaling. We determined that SMAD3 and FOXH1 were specifically bound to the *Gsc* and *Eomes* promoters in the pluripotent ESC state, as determined by ChIP-PCR analysis with SMAD isoform-specific antibodies in wild-type versus S3KO and *Foxh1*^−/^^−^ ESCs (Supplemental Fig. S5A). Compared with wild-type mESCs, *Foxh1*^−^^/−^ mESCs showed a partial loss of SMAD3 binding to the *Eomes* promoter and a complete absence of SMAD3 binding to the *Gsc* and *Foxa2* promoters. In contrast, FOXH1 binding to these regions showed only a limited decrease in S3KO mESCs compared with wild-type ESCs (Supplemental Fig. S5A). These results suggested that FOXH1 is the main driver of basal signal-independent SMAD3 binding to these promoters.

The binding of SMAD3 to the *Gsc*, *Eome*, and *Foxa2* promoters was not significantly inhibited in SMAD4 knockout ESCs compared with wild type or by cell treatment with SB (Supplemental Fig. S5A,B), providing further evidence that the basal binding of SMAD3 in pluripotent ESCs was independent of SMAD4 and endogenous Nodal signals. Comparing day-3 EBs derived from WT and SMAD4 knockout cells treated with Activin, the absence of SMAD4 did not decrease the interaction of SMAD3 with the *Gsc* and *Eomes* promoters but inhibited the interaction of SMAD2 with both the promoters and the enhancers of these genes (Supplemental Fig. S5C). The joint binding of FOXH1 and SMAD3 to common sites was also manifested at the genome-wide level, as determined by FOXH1 ChIP-seq tag density analysis within 3 kb of SMAD3 binding peaks in pluripotent ESCs (Supplemental Fig. S5D). Notably, SMAD2 bound poorly to the *Eomes* promoter in wild-type ESCs but strongly in SMAD3 knockout ESCs under pluripotency conditions (Supplemental Fig. S5E), showing that SMAD2 can take the place of SMAD3 in binding to this promoter when SMAD3 is absent.

Collectively, these results suggest that the pioneer factor FOXH1 binds to regulatory regions of mesendoderm differentiation genes in the absence of Nodal/Activin signaling and recruits SMAD3 to these promoters in preference over the fourfold more abundant SMAD2. Nodal/Activin signaling induces formation of a SMAD2:SMAD4 complex that joins the prebound SMAD3 and FOXH1 complex at these promoters, triggering gene expression.

### The E3 insert promotes Nodal signaling

**Figure 6.**
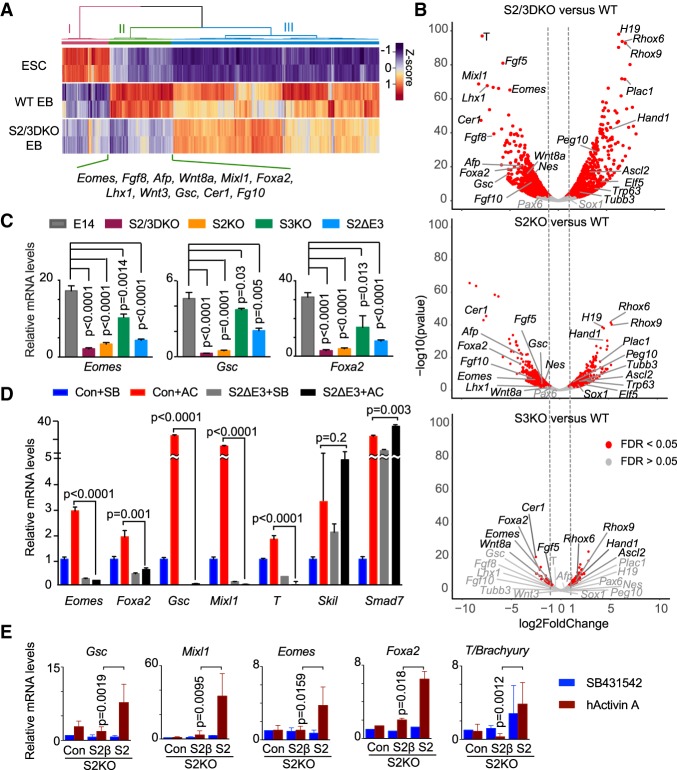
The SMAD2 E3 insert promotes Nodal-dependent mesendoderm gene expression. (*A*) Heatmap of the top 500 genes with the highest variance of expression between wild-type mESCs and day-4 EBs RNA-seq transcriptomic profiles and expression of these genes in day-4 S2/3DKO mESCs. Three classes are highlighted: (I) Genes expressed in mESCs and down-regulated in EBs; (II) genes up-regulated in wild-type EBs but not in S2/3DKO EBs, which include many mesendoderm differentiation genes; (III) genes up-regulated in wild-type as well as S2/3DKO EBs. Two biological replicates at each condition were analyzed. (*B*) Volcano plot of RNA-seq transcriptomic data of day-4 EBs derived from S2/3DKO, S2KO, S3KO cells, compared with wild-type EBs. Each red dot represents a gene that was differentially under- or overexpressed (false discovery rate <0.05) in the SMAD-deficient cells compared with wild type. Representative lineage specification genes for mesendoderm (*T/Brachyury*, *Foxa2*, *Eomes*, *Mixl1*, *Gsc*, *Lhx1*, *Afp*, *Cer1*, *Fgf8*, *Fgf10*, *Fgf5*, and *Wnt8a*), ectoderm (*Nes*, *Pax6*, *Sox1*, *Tubbe*, and *Trp63*), and extra-embryonic fates (*H19*, *Rhox6*, *Rhox9*, *Plac1*, *Peg10*, *Ascl2*, and *Elf5*) are highlighted. Two biological replicates for each condition were analyzed. (*C*) qRT-PCR analysis of representative mesendoderm genes (*Eomes*, *Gsc*, *Foxa2*) in day-4 EBs derived from wild-type, S2/3DKO, S2KO, or S3KO cells. *N* = 3; error bars represent SD, and *P*-values were calculated by *t*-test. (*D*) qRT-PCR analysis of the indicated mesendoderm genes and pathway feedback genes in day-3 EBs from wild-type or S2ΔE3 cells treated with SB or Activin for 2 h. Experiment performed in triplicate, one representative set of results is shown. Error bars represent SD and *P*-values were calculated by *t-*test. (*E*) qRT-PCR analyses of representative mesendoderm genes in day-3 EBs derived from *Smad2*^–/–^ mESCs expressing HA-tagged human SMAD2, HA-tagged human SMAD2β, or empty vector as control (*Con*). Cells were treated with SB or Activin for 2h. mRNA levels of each gene are expressed relative to the SB condition in the control cells. *N* = 3, biological replicates; error bars represent S.D. Two-tailed Mann–Whitney test.

Next, we investigated whether the E3 insert limits or enhances Nodal activation of mesendoderm genes. Transcriptomic profiling of wild-type mESCs, wild-type and S2/3DKO day-4 EBs defined several classes of differentiation-associated gene expression events (Fig. 6A). Class I includes genes that were expressed in wild-type mESCs and down-regulated both in wild-type and S2/3DKO EBs. Class II includes genes that were up-regulated in wild-type EBs but not in S2/3DKO EBs; this Smad2/3-dependent class includes many mesendoderm differentiation genes (Fig. 6A). Class III includes genes that were up-regulated in wild-type as well as S2/3DKO EBs. Volcano plots of day-4 EB RNA-seq data from S2/3DKO, S2KO, and S3KO mESCs showed the relative dependence of differentiation-associated genes on SMAD2 and SMAD3. Compared with wild-type EBs, S2/3DKO EBs were markedly deficient in the expression of *Gsc*, *Eomes*, *Foxa2*, *T/Brachyury*, *Mixl1*, *Lhx1*, *Afp*, *Cer1*, *Fgf8*, *Fgf10*, *Fgf5*, and *Wnt8a* (Fig. 6A,B), S2KO EBs were also strongly deficient in the expression of these genes, whereas S3KO cells were only marginally deficient. In each case, the diminished expression of mesendoderm genes was accompanied by a proportional gain in the expression of extra-embryonic cell fate genes (*H19*, *Rhox6*, *Rhox9*, *Plac1*, *Peg10*, *Ascl2*, and *Elf5*) (Fig. 6B). In all, these findings are concordant with reports that SMAD2 is essential for embryonic development, whereas SMAD3 has a more limited role in this context (Nomura and Li 1998; Waldrip et al. 1998; Weinstein et al. 1998; Ashcroft et al. 1999; Yang et al. 1999), with loss of SMAD2 and SMAD3 enabling the emergence of extra-embryonic cell fates (Senft et al. 2018).

To determine the contribution of the SMAD2 E3 insert, we performed quantitative reverse transcriptase PCR (qRT-PCR) analysis of *Eomes*, *Gsc*, *Foxa2* as representative mesendoderm differentiation genes in wild-type, S2ΔE3, and SMAD-deficient day-4 EBs. The results showed an intermediate loss in the expression of these genes in S2ΔE3 cells, compared to the losses observed in S2KO mESCs and S3KO mESCs (Fig. 6C). S2ΔE3 EBs showed a diminished induction of *Eomes*, *Foxa2*, *Gsc*, *Mixl1*, and *T*, and an intact or increased induction of *Smad7* and *Skil* in response to Activin (Fig. 6D). Moreover, expression of exogenous SMAD2 in S2KO mESCs rescued the Activin response of *Eomes*, *Foxa2*, *Gsc*, *Mixl1*, and *T*, whereas expression of exogenous SMAD2β was poor at rescuing these responses (Fig. 6E; Supplemental Fig. S6A). Thus, the E3 insert is required for maximal induction of mesendoderm differentiation genes by SMAD2.

### The SMAD2 E3 insert promotes early mouse development

**Figure 7.**
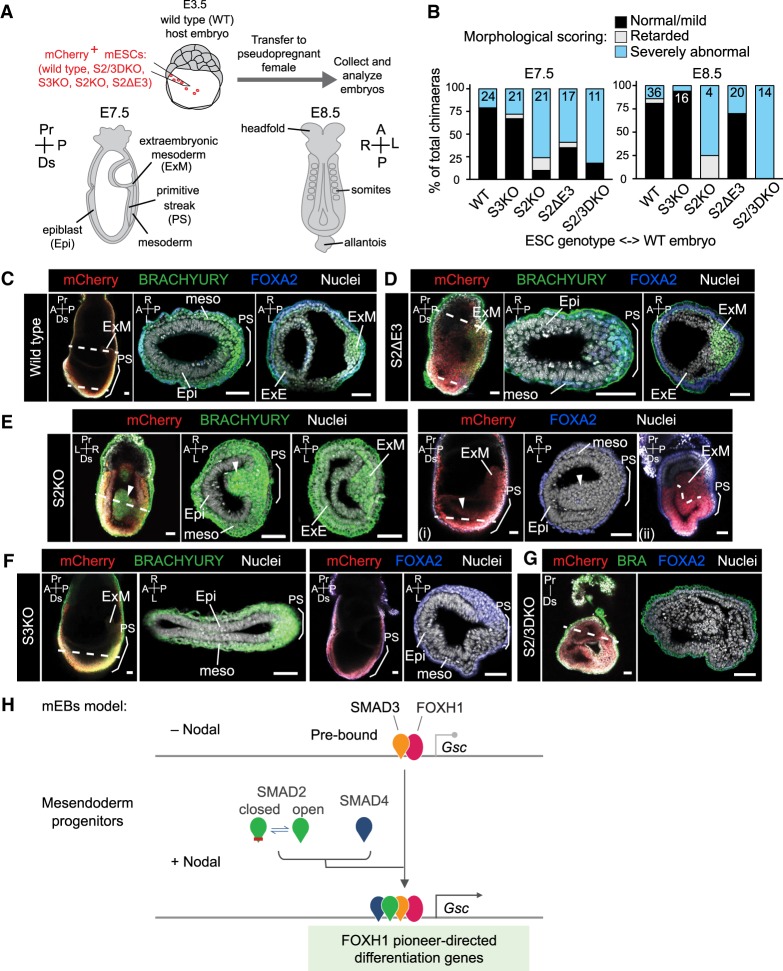
The SMAD2 E3 insert promotes early mouse development. (*A*) Schematic of embryo chimera generation by injecting mESCs expressing a constitutive mCherry marker into wild-type E3.5 blastocysts. Embryos were transferred to pseudopregnant females and dissected at E7.5 and E8.5 to assess development. (*B*) Chimeras, generated by injecting either WT or S3KO, S2KO, S2ΔE3, S2/3DKO, mESCs into WT E3.5 blastocysts were dissected at E7.5 and E8.5 and categorized based on gross morphology as normal/mild defects, developmentally retarded or severely abnormal. At E7.5, a small fraction of WT chimeras displayed small clumps of cells in the amniotic cavity, possibly as an artifact of the microinjection and hence were scored as abnormal. Numbers shown within the bars represent the number of chimeric embryos obtained and scored. (*C*–*G*) Confocal sagittal optical sections of whole-mount immunostained chimeric embryos and cryosections of representative embryos. Dashed lines indicate approximate plane of section. Nuclei were stained with Hoechst. Note, mCherry fluorescence, marking mESC progeny, was diminished postfixation of whole-mount imaging and was not clearly observed after cryosectioning. Arrowheads in panel *F* mark abnormal cell masses protruding into the cavity. Dashed line in the last panel of panel *F* marks the presumptive boundary between the epiblast and extraembryonic mesoderm. Brackets demarcate the primitive streak (PS). HF, headfold; NT, neural tube; Al, allantois; Am, amnion; Epi, epiblast; ExM, extraembryonic mesoderm; ExE, extraembryonic ectoderm; meso, mesoderm; A, anterior; P, posterior; Pr, proximal; Ds, distal; L, left; R, right. Scale bars, 50 µm. (*H*) Model of Nodal/SMAD signaling in the activation of differentiation genes and in mouse mesendoderm progenitors. Mesendoderm differentiation genes (e.g., *Gsc*) are bound by the pioneer factor FOXH1, which recruits SMAD3 to regulatory elements in the absence of Nodal signals, whereas the unique E3 insert of SMAD2 conditionally limits DNA-binding activity and allows SMAD2 to remain poised for Nodal/Activin-driven binding of SMAD4 from signal transduction to the nucleus. Thus, a basal SMAD3–FOXH1 complex primes mesendoderm differentiation genes for regulation, whereas signal-driven SMAD2:SMAD4 complexes join SMAD3 and FOXH1 to trigger transcriptional activation.

To assess the developmental potential of mESCs in vivo as a function of their ability to express SMAD2, SMAD2β, and/or SMAD3, we microinjected wild-type, S2/3DKO, S3KO, S2KO, and S2ΔE3 mESCs labeled with mCherry into wild-type mouse blastocysts to generate chimeric embryos (Fig. 7A; Wang et al. 2017). Chimeras were dissected at embryonic days (E) 7.5 and E8.5, corresponding to midgestation, and analyzed for the contribution of mCherry^+^ cells to major compartments (Supplemental Fig. S7A,B).

Chimeric embryos containing wild-type cells generally developed normally, while chimeric embryos containing mutant cells exhibited a variety of morphological defects at E7.5 and E8.5, around the time of gastrulation (Fig. 7B; Supplemental Fig. S7C–E). In agreement with previous findings (Zhu et al. 1998; Ashcroft et al. 1999; Datto et al. 1999), the majority of chimeric embryos containing S3KO cells were morphologically normal, established a T/BRACHYURY-positive (*T*^+^) primitive streak (PS), PS-derived embryonic and extraembryonic mesoderm, and FOXA2^+^ definitive endoderm and/or axial mesoderm precursors (Fig. 7B,C,F,G; Supplemental Fig. S7A–E). In contrast, a large proportion of chimeric embryos containing S2KO or S2ΔE3 cells exhibited morphological abnormalities including folding of the epiblast epithelium, angular distortions of the typically rounded and cylindrical epiblast, and an indistinct embryonic–extraembryonic boundary (Fig. 7B,D,E; Supplemental Fig. S7C–F,H). In S2KO chimeric embryos, prominent masses of cells within the amniotic cavity were observed (Fig. 7E). Despite these abnormalities, S2KO or S2ΔE3 chimeric embryos formed *T*^+^ PS and specified embryonic and extraembryonic mesoderm lineages, possibly by contribution or rescue by endogenous wild-type cells (Fig. 7D,E; Supplemental Fig. S7G,H), as reported in SMAD2 mutant embryo (Vincent et al. 2003).

In some S2KO (Fig. 7E) and the majority of S2ΔE3 chimeric embryos (Fig. 7D; Supplemental Fig. S7H) there was a notable increase in extraembryonic mesoderm, which could impose elevated force on the adjacent cell layers and underlie the aberrant epiblast morphology. Compared with controls, S2KO cells did not efficiently give rise to definitive endoderm (Tremblay et al. 2000; Dunn et al. 2004), but we observed FOXA2^+^ S2ΔE3 cells within the PS of mutant embryo chimeras (Fig. 7E; Supplemental Fig. S7E–H). As the primary abnormalities observed within S2ΔE3 chimeric embryos were associated with the extraembryonic mesoderm rather than the embryo-proper, by E8.5 most embryos appeared morphologically normal, although a number of embryos had kinked neural tubes possibly resulting from the initial distortion of the epiblast (Supplemental Fig. S7B,E).

In the presence of a functional *Smad2* allele, SMAD3 is not necessary for early development (Nomura and Li 1998; Heyer et al. 1999; Dunn et al. 2004, 2005). However, reducing the dose of *Smad3* in the absence of SMAD2 causes severe developmental defects (Dunn et al. 2004). In keeping with this, S2/3DKO chimeric embryos exhibited the most severe defects. At E7.5 and E8.5, embryos containing a high proportion of S2/3DKO mESC progeny formed round masses of highly folded cell layers encompassed by an expanded parietal yolk sac (Fig. 7B,C; Supplemental Fig. S7A–E,I,J). We also noted a high number of pyknotic nuclei and mCherry^+^ cell debris, suggesting extensive death of S2/3DKO cells. In most embryos, no clear A-P axis or expression of T or FOXA2 was discerned, implying that embryos were not undergoing gastrulation. In sum, the most severe phenotypes were observed in S2/3DKO chimeric embryos, followed by S2KO*,* and S2ΔE3 chimeric embryos. These observations collectively suggest that the E3 insert supports the mesoderm-inducing activity of SMAD2.

## Discussion

The present work defines distinct roles for SMAD2 and SMAD3 in the regulation of differentiation genes with FOXH1 as DNA binding partner in mesendoderm progenitors. Here, SMAD2 and SMAD3 cooperate as mediators of gene expression, with SMAD2 serving as a conditional DNA binding protein and classic signal-driven transcriptional regulator, and SMAD3 with the pioneer factor FOXH1 binding to target promoters and marking these sites for incorporation of signal-driven SMAD2:SMAD4 complexes (Fig. 7H). The basis for the distinct behavior of SMAD2 and SMAD3 is illuminated by our evidence that SMAD2 has DNA binding activity that is determined by the ensemble of conformations adopted by a unique structural element, the E3 insert.

### Conditional DNA-binding activity of SMAD2

We show that properly folded SMAD2 interacts with DNA. This finding argues against the long-held notion that this crucial mediator of TGF-β transcriptional responses cannot bind DNA. The MH1 domains of SMAD2 and SMAD2β specifically interact with double-stranded DNA oligonucleotides containing canonical GTCTG and 5GC SMAD binding sites. The basis for the DNA-binding activity of SMAD2 is revealed by our X-ray crystal structure analysis of the canonical core MH1 fold of SMAD2β, and the characterization of the NMR conformations adopted by the E3 insert that protrudes from this fold. The apparent affinity of the SMAD2 MH1 domain for GTCTG and 5GC DNA probes is fourfold lower than that of SMAD2β, SMAD3, and SMAD4, conceivably reflecting a mixture of open and closed DNA-binding conformations that are suggested by NMR relaxation analysis of the E3 insert.

The X-ray crystal structure of the SMAD2β MH1 domain bound to the GTCTG DNA motif shows that the overall fold and DNA-binding mode of this isoform conforms to the canonical fold and DNA-binding characteristics of other SMAD proteins (Shi et al. 1998; Chai et al. 2003; BabuRajendran et al. 2010; Martin-Malpartida et al. 2017), with only small differences in the shape of the bound DNA due to the presence of the fifth base pair of the specific GTCTG SBE motif used in this structure. NMR analysis of the SMAD2 MH1 domain shows that the E3 insert is flexible but not disordered. The pattern of NOEs detected for the insert indicates the presence of two short helices (α1′ and α2′) protruding from the canonical MH1 domain structure as a necklace anchored between the β1 strand and the loop that precedes the DNA-binding β2–β3 hairpin. The E3 insert populates an ensemble of conformations that differ in their relative orientation to the MH1 domain fold. The conserved core of the MH1 domain restricts the possible orientations that the E3 insert can sample in the presence or absence of DNA. These orientations are conditioned by the proximity of the antiparallel β1–β5 and β2–β3 hairpins of the MH1 domain, which need to remain structured in order to maintain the MH1 fold. In the open conformation, the start of the E3 insert is in the proximity of loop 7, which connects the 3_10_ helix to the β5 strand. This orientation of the E3 insert allows the DNA-binding hairpin, the preceding loop and the insert itself to contact DNA, as reflected by the chemical shift differences in these residues in the presence of DNA. Other conformations of the E3 insert occlude the DNA-binding hairpin and bar it from interacting with DNA. In these closed conformations the E3 insert covers a hydrophilic area, which would limit the solubility of recombinant SMAD2 and explain the failure of this protein to bind DNA in previous reports. The transition between open and closed soluble conformations involves a rotation along the β1 strand, which behaves as a hinge that ensures the interconversion of conformations without disrupting the MH1 fold (Supplemental Movie S1). SMAD2-SMAD4 interactions or SMAD2 posttranslational modifications might influence the equilibrium between the open and closed conformations of the E3 insert in vivo, a question for future investigation.

### Distinct roles of SMAD2 and SMAD3 in TGF-β signaling

Our results reveal that SMAD2 and SMAD3 play nonequivalent, complementary roles in Nodal activation of differentiation genes in mouse mesendoderm progenitors. FOXH1 in these cells acts as a pioneer factor prebound to *cis*-regulatory elements of mesendoderm differentiation genes linke *Gsc* and *Eomes* in the absence of Nodal signals. FOXH1 recruits SMAD3 to these promoters in the absence of Nodal signal, establishing a basal complex that is joined by SMAD2 and SMAD4 under Nodal signaling. The binding of SMADs to target promoters was previously unappreciated, but various reported observations are in line with our present finding. SMAD protein shuttle between the cytoplasm and the nucleus even in the absence of TGF-β signal (Kurisaki et al. 2001; Xu et al. 2002). FOXH1 can directly bind to the MH2 domain of SMAD2 and SMAD3 (Liu et al. 1997). FOXH1 is a pioneer factor that binds to target regulatory regions to subsequently recruit additional factor, therefore it may directly recruit SMAD3 to these sites as this protein cycles through the nucleus under basal conditions.

SMAD3 is expressed at fourfold lower levels than SMAD2 in mesendoderm progenitors, yet SMAD3 is preferentially recruited by FOXH1 while SMAD2 acts as a classic signal-dependent mediator that forms a complex with SMAD4 and the two proteins join the promoter-bound SMAD3 and FOXH1 in response to Nodal signals. The preferential recruitment of SMAD3 by FOXH1 is not necessarily due to a higher affinity for SMAD3 over SMAD2 but possibly to the superior ability of SMAD3 to contact DNA. It is conceivable that the capacity of SMAD2 to inhibit its own DNA-binding activity by closed conformations of the E3 insert prevents SMAD2 from competing with SMAD3 for FOXH1-mediated recruitment to these promoters. The restriction imposed by the E3 loop on DNA binding, together with a previously identified restriction on SMAD2 interaction with nuclear import factors (Kurisaki et al. 2001), may preserve SMAD2 for signal-dependent recruitment of SMAD4. In contrast, FOXH1 constitutively recruits SMAD3 to regulatory elements of differentiation genes and primes these sites for further incorporation of Nodal-driven SMAD2:SMAD4 complexes to achieve transcriptional activation (Fig. 7H).

Our evidence that SMAD2 and SMAD3 play complementary and mutually compensatory roles is in line with the observed phenotypes of SMAD2-deficient and SMAD3-deficient embryos (Nomura and Li 1998; Heyer et al. 1999; Dunn et al. 2004, 2005). While SMAD2 acts as the main transducer of Nodal receptor signals and its loss causes marked developmental defects in culture and in embryo, the combined loss of SMAD2 and SMAD3 causes the most profound developmental defects. In the presence of a functional *Smad2*, *Smad3* is largely dispensable for early development (Nomura and Li 1998; Heyer et al. 1999; Dunn et al. 2004, 2005). We show that when SMAD3 is absent, SMAD2 can take its place as a FOXH1-recruited factor in the basal state. However, SMAD2 is less efficient in this role than is SMAD3, and *Smad3* mutant mesendoderm progenitors show differentiation defects in culture and developmental defects in embryos. The present findings on the nonequivalence and complementary nature of SMAD2 and SMAD3 may also apply to other contexts in which TGF-β regulation of differentiation is directed by pioneer transcription factors that are prebound to the chromatin and provide a template for rapid activation of specific genes by the TGF-β signal transduction pathway.

## Materials and methods

### Protein production and cloning

Human: SMAD2 (Q15796-1), MH1 domain (Pro6-Val180) and full-length (Pro6-467), SMAD2β (Q15796-2), Pro10-Val144, and Pro10-437, SMAD4 (Q13485), Pro10-Gly140 and Pro10-Asp552 and SMAD3 (P84022) Pro10-Pro136 (MH1 domain). *Xenopus*: SMAD2 (NP_001084964), Pro10-Val180 (MH1 domain). All fragments were amplified by PCR using DNA templates (Thermo Fisher Scientific) with optimized codons for bacterial expression. Single- and double-point mutations were introduced using the QuikChange II system (Agilent, 200521). Fragments were purified using PureLink PCR kit (Invitrogen) and incorporated to the plasmid of choice by recombination (RecA recombinase, New England Biolabs). All sequences were confirmed by DNA sequencing (GATC Biotech). Specific details of the purification are provided as Supplemental Methods.

### Electrophoretic mobility shift assay (EMSA)

Duplex DNAs were annealed using complementary single-strand HPLC purified DNAs. DNAs were mixed at equimolar concentrations (1 mM) in 20 mM Tris pH7.0 and 10 mM NaCl, heated at 90°C for 3 min and cooled down to room temperature during 2 h. DNAs (with and without Cy5-fluorophores) were purchased from Biomers or Metabion.

Binding reactions were carried out for 30 min at 4°C in 10 μL of binding buffer (50 mM Tris pH 8, 150 mM NaCl, 2mM TCEP, 10% Glycerol). A fixed concentration of 5′-end Cy5-labeled (Biomers, Germany) duplex DNA (7.5 nM) was incubated with increasing amounts of SMAD MH1 domains or with full-length proteins. Electrophoresis were performed in nondenaturing 4.5 and 8% native polyacrylamide gels (1.5-mm thick), prepared with 30% acrylamide/bis-acrylamide, 37.5:1 solution (Bio-Rad). The gels were run for 1 h in TG buffer at 90 V at 4°C. None of the buffers contain EDTA. The gels were exposed to a Typhoon imager (GE Healthcare).

### X-ray crystallography

High-throughput crystallization screening and optimization experiments were performed at the HTX facility of the EMBL Grenoble Outstation (Zander et al. 2016). Human SMAD2β was concentrated to 5 mg/mL prior to the addition of the annealed DNAs (Metabion) dissolved in 20 mM Tris-HCl pH 7, 10 mM NaCl. The final protein DNA molar ratio was 1:1. Specific details of the X-ray crystallography are provided as Supplemental Methods.

### NMR chemical-shift assignment and perturbation experiments

NMR data corresponding to both SMAD2 isoforms were recorded on a Bruker Avance III 600-MHz spectrometer equipped with a quadruple (^1^H, ^13^C, ^15^N, ^31^P) resonance cryogenic probe head and a z-pulse field gradient unit at 298 K. Backbone ^1^H, ^13^C, and ^15^N resonance assignments were obtained by analyzing the 3D HNCACB and HN(CO)CACB experiment pair (Solyom et al. 2013). Experiments were acquired as band-selective excitation short-transient-type experiments (BEST) with TROSY and nonuniform sampling (NUS) (Orekhov and Jaravine 2011). ^15^N-Edited 3D NOESY and 2D NOESYs at different mixing times were recorded to assign proton resonances. Chemical shifts have been deposited in the Biological Magnetic Resonance Data Bank, entries BMRB:27742 and BMRB:27743 (corresponding to SMAD2 and SMAD2β). For the screening search of protein expression, HSQC experiments were recorded using a Non-Uniform Sampling (NUS) acquisition strategy to reduce experimental time and increase resolution. Relaxation measurements were acquired using standard pulse sequences (Barbato et al. 1992). Spectra were processed with NMRPipe (Delaglio et al. 1995) and MddNMR (multidmensional decomposition and compressed sensing algorithms for NMR) (Orekhov and Jaravine 2011) and assigned with CARA (http://cara.nmr.ch/doku.php).

### SAXS data

Data were collected on samples of SMAD2 at protein concentrations of 1, 3, and 5 mg/mL and SMAD2β of 1.3, 3.8, and 6.8 mg/mL. All samples were concentrated in 20 mM Tris buffer, 150 mM NaCl, and 2 mM TCEP, pH 7.2. Data were acquired at Beamline 29 (BM29) at the European Synchrotron Radiation Facility (ESRF; Grenoble, France). Protein samples were centrifuged for 10 min at 10,000*g* prior to data acquisition. Experiments on BM29 were collected at an energy of 12.5 keV and data were recorded on a Pilatus 1M detector at 10°C. For each sample and buffer, 10 exposure frames of 1 sec were collected, and the exposure set was combined during data reduction to produce each SAXS curve. Buffer subtraction was performed after data reduction. Image conversion to the 1D profile, data reduction, scaling, and buffer subtraction were done by the software pipeline available at the BM29 beamline. Further processing was done with the ATSAS software suite and Scatter (Franke et al. 2017). Guinier plot calculation (for the estimation of the radius of gyration Rg) was performed with PRIMUS, included in the ATSAS suite, using low *q* regions (*q*_max_ × Rg < 1.3). SMAD2 conformations were generated using the Rosetta modeling software suite, using the RosettaCM application (Song et al. 2013) and starting from the MH1 crystal structures of SMAD2β determined in this work. In all cases, DNA and water molecules were removed and secondary structure elements were restrained, except for the flexible N and C-terminal tails, the flexible Exon3, and G-loop regions. Five thousand conformers were simulated in order to generate sufficient conformational sampling. For SMAD2 MH1, the E3 insert secondary structure determined by NMR, (83–86 and 91–98 helices) were built using Modeller (Eswar et al. 2006). Theoretical SAXS curves were calculated using CRYSOL (Svergun et al. 1995) and fitted to the experimental data using the ensemble optimization method as implemented in ATSAS (Bernadó et al. 2007). The chi-squared metric for *N* data points was calculated using the equation:

$$X^{2}=\frac{1}{N}\sum_{i=1}^{N}\frac{{\left[I^{\mathrm{calc}}(q_{i})-I^{\mathrm{obs}}(q_{i})\right]}^{2}}{\sigma_{i}}.$$

### Cell line maintenance and differentiation

ES-E14TG2a.4 *Mus musculus* embryonic stem cells were maintained on plates coated with gelatin (0.1%, Millipore, ES-006-B) in LIF-supplemented medium at 37°C with 5% CO_2_. EB formation and differentiation were carried out as described by the supplier (ATCC). *Foxh1*^–/–^ mECSs were a gift from L. Attisano and J. Wrana (Izzi et al. 2007).

### Genome-editing with CRISPR/Cas9

Annealed sgRNA oligos were cloned into pSpCas9 (BB)-2A-GFP or pSpCas9 (BB)-2A-puro Addgene vectors (Ran et al. 2013) and transiently transfected into E14TG2a mouse ES cells with Lipofectamine 3000 (Life Technologies). Single cells after sgRNA transfection were seeded onto irradiated MEF feeder for increased viability. Mutant clones were verified by PCR, TA-cloning and sequencing. The sgRNA target sequences are as following: *Smad2*: TTCACCACTGGCGGAGTGAA; *Smad3*: GACGGGGCAGTTGGACGAGC; and SMAD2 exon 3: TGCTGACCCGTTGGGTG and GGACCCTAGAGACCGCGT.

### Plasmids, lentivirus, and chemicals

Lentiviral infections and plasmid transfections were performed as previously described (Xi et al. 2011). To generate plasmids for doxycycline-inducible vectors, the ORFs of SMAD2 and SMAD2β were cloned into pLVX-Tight-Puro vector (Clontech), respectively, and HA- tag was added at the N-terminal accordingly. In addition, the CMV promoter present in plasmid pLVX-Tet-On was replaced with a pGK promoter to avoid silencing in mESCs.

### qRT-PCR analysis

RNA extraction and analysis were done as previously described (Wang et al. 2017). qRT-PCR oligonucleotide primers were as previously reported (Wang et al. 2017) or listed as Supplemental Table S2.

### Chromatin immunoprecipitation

For chromatin immunoprecipitation (ChIP)-qPCR and ChIP-seq, mouse ES cells and EBs were collected at indicated time points. Some cells had been treated with human recombinant Activin A (2 nM; R&D Systems) for 2 h or SB431542 (SB, 10 µM, Tocris) for 2–4 h, as indicated. ChIP was performed as previously described (Xi et al. 2011; Wang et al. 2017). For ChIP-qPCR, immunoprecipitated DNA was analyzed by qRT-PCR, and the amplification product was expressed as percentage of the input, or then normalized to the control experiment for each condition. The PCR primer pairs used to amplify the unrelated control, distal enhancer, and promoter regions of indicated genes were as previously described (Wang et al. 2017) or as follows: *Gsc* PP, 5′-GTTGGGAATTGTCCCACTCT-3′ (forward) and 5′-GGAGGAGGGAGTTCGGA-3′ (reverse); *Eomes* PP, 5′-CCCAACTGGCCTTTATAACCA-3′ (forward) and 5′-CTCTCCCAACTGCATGCTTTA-3′ (reverse); *Foxa2* PP: 5′-TGTGTCTGTCAGTTGGTCTATTC-3′ (forward) and 5′-CAGCTGGGAGCACAATCAAAG-3′ (reverse); *Smad7* PP, 5′-TTGAAACAGACAGCGATCTCC-3′ (forward) and 5′-GGTTAGTGGCCCGATTTAGAC (reverse); *Smad7*_DE: 5′-TAGGCTCCGCAAGGTTAGA (forward)-3′ and 5′-TGTGGGAGCCCAAGTTTATG (reverse). Antibodies used were against SMAD2 (5339S, Cell Signaling Technology), SMAD3 (9523S, Cell Signaling Technology), SMAD4 (7966X, Santa Cruz and 40759, Abcam), and FOXH1 (49133, Abcam).

### Immunoblotting and immunoprecipitation

Cell pellets were lysed with RIPA buffer (Cell Signaling) and protein concentrations were determined using the BCA Protein Assay Kit (Pierce). The Nuclear Complex Co-IP Kit (Active Motif, 54001) was used for immunoprecipitation. Whole-cell lysate was used for immunoprecipitation of SMAD4. Cell lysate was further diluted in 50 mM Tris-HCl PH 8.0, 120 mM NaCl, 1 mM EDTA, 0.5% NP40 supplemented with protease inhibitor and phosphatase inhibitor. Proteins were separated by SDS-PAGE using Bis-Tris 4%–12% gradient polyacrylamide gels in the MOPS buffer system (Life Technologies) and transferred to nitrocellulose membranes (BioRad) according to standard protocols. Membranes were immunoblotted with antibodies against SMAD2 (5339S, Cell Signaling Technology), SMAD2/2β (3103S, Cell Signaling Technology), SMAD3 (9523S, Cell Signaling Technology), SMAD4 (7966X, Santa Cruz and 40759, Abcam and 38454, Cell Signaling Technology), and γ-Tubulin (T6074, Sigma-Aldrich) in Odyssey-TM blocking buffer (LI-COR). Following incubation with primary antibody, membranes were washed and probed with IRDye 800CW donkey-anti-mouse IgG (LI-COR) or IRDye 680RD goat-anti-rabbit IgG (LI-COR) secondary antibody and imaged using the LI-COR Odyssey system. All western immunoblots were performed at least twice. γ-Tubulin was used as a loading control for all experiments.

### Cell fractionation assay

The Nuclear Complex Co-IP Kit (Active Motif, 54001) was used for cell fractionation assay following the manufacturer's protocol. Briefly, 1 × 10^7^ cells were collected, suspended in hypotonic buffer, and incubated on ice for 15 min. Detergent was added, and cell suspension was centrifuged. The supernatant was collected as cytoplasmic fraction. The pellet was suspended in DNA digestion buffer and incubated on ice for 90 min. EDTA was added to stop the reaction, the suspension was centrifuged, and the supernatant was collected as nuclear fraction.

### Data analysis

RNA-seq or ChIP-seq data analysis were done as previously described (Wang et al. 2017). For mapping and visualization, single end (50 bp) or paired-end (50/50 bp) FASTQ reads were mapped to mouse genome mm10 (GRCm38, 2011) with Bowtie2 with default filtering criteria (Langmead and Salzberg 2012). Samtools was used to manipulate .sam and .bam files (Li et al. 2009). Tag directories, visualization in UCSC genome browser, and downstream analyses were performed using the HOMER suite (Heinz et al. 2010). To visualize ChIP-seq data, BAM files were converted to TDF file by IGV Tools 2.3.32 (Robinson et al. 2011) using the command “igvtools count -z 5 -w 25 -e 250”, specifying the coverage window size to be 25 bp and average fragment size of 250 bp. The relative abundance of *Smad2* and *Smad2β* transcripts between day 0 and day 4 of ESC to EB differentiation was determined based on the GSE70486 data set. For each read pair that mapped to the mouse *Smad2* locus was analyzed for the presence or absence of exon 3-encoded sequence.

### Statistical analysis

Quantitative data are expressed as mean ± standard deviation. Statistical significance was determined using a two-tailed Mann–Whitney test or *t*-test using Prism 7 software (GraphPad Software) unless otherwise indicated.

### Generation of chimeric embryos

mCherry expressing single mESC colonies were picked and micro-injected 3 d after culture on MEF feeder layers. A total of 10–15 mESCs from each group were injected into E3.5 blastocysts (C57BL/6N Taconic) as published (Wang et al. 2017). Injected blastocysts were implanted into the uterine horns (10 embryos per horn) of E2.5 pseudopregnant females using standard protocols. Chimeric embryos were recovered at E7.5 and E8.5 and analyzed as described in the Supplemental Information. Specific details of the generation of chimeric embryos are provided as Supplemental Methods.

## Data and software availability

All RNA-seq and ChIP-seq data were deposited in the Gene Expression Omnibus database under accession number GSE125116. Chemical shifts and SAXS data have been deposited in the Biological Magnetic Resonance Data Bank, entries BMRB:27742 and BMRB:27743 and SASDG35/SASDG45 for SMAD2 and SMAD2β MH1 domains. The densities and coordinates of the SMAD2β MH1 complex bound to DNA have been deposited in the Protein Data Bank, accession code PDB:6H3R.

## Competing interest statement

J.M. is a science advisor and owns company stock of Scholar Rock. The remaining authors declare no competing interests.

## Supplementary Material

Supplemental Material
