# Supplementary material for: Structural basis for distinct roles of SMAD2 and SMAD3 in FOXH1 pioneer-directed TGF-β signaling
Source: Genes Dev. 2019 Nov 1;33(21-22):1506–24. doi: 10.1101/gad.330837.119 (PMC6824466; doi:10.1101/gad.330837.119)
Supplement: Supplemental Material [file supp_33_21-22_1506__index.html]

Structural basis for distinct roles of SMAD2 and SMAD3 in FOXH1 pioneer-directed TGF-β signaling — Supplemental Material 

# Structural basis for distinct roles of SMAD2 and SMAD3 in FOXH1 pioneer-directed TGF-β signaling

## Supplemental Material

- Supplemental\_Material.pdf
- Supplemental\_Movie\_S1.mp4
